# Cyclosporine: A Commentary on Brand versus Generic Formulation Exchange

**DOI:** 10.1155/2011/480642

**Published:** 2011-11-17

**Authors:** A. K. Singh, S. S. Narsipur

**Affiliations:** Division of Nephrology, Department of Medicine, Upstate Medical University, Syracuse, NY 13210-2342, USA

## Abstract

The evidence for conversion from brand name to generic equivalent cyclosporine is conflicting. Cyclosporine is a narrow therapeutic-range drug for which small variations in exposure may have severe clinical consequences for transplant patients. There is currently a lack of comparative outcome data relating to the pharmacokinetics of the reference formulation, Neoral, and generic formulations in transplant recipients. A major common concern is the potential inability to attain similar trough levels, an issue that can be easily corrected by ongoing therapeutic drug monitoring to ensure that the new steady state falls within an intended target range. Prospective clinical studies investigating the efficacy and safety of generic formulations in both *de novo* and long-term transplant patients are also awaited. Until further evidence is available on the conversion of transplant patients to or between generic formulations of cyclosporine, any transfer to a different cyclosporine formulation should be undertaken with close supervision. The best available information to date, however, does not support the frequently held but unsubstantiated belief that generic preparations of immunosuppressive drugs are not as effective as brand names or that conversion from brand to generic is associated with significant danger. This paper attempts to initiate a discussion of these issues.

## 1. Background

The discovery of cyclosporine was a landmark in the history of solid organ transplantation. It was initially isolated from the fungus *Tolypocladium inflatum* from a soil sample obtained by Sandoz scientists at Hardangervidda, Norway in 1970 [1]. Cyclosporine is a cyclic nonribosomal peptide of 11 amino acids and contains a single D-amino acid, which is rarely encountered in nature. The drug exhibits very poor solubility in water and is lipophilic. As a consequence, suspension and emulsion forms of the drug have been developed for oral administration and for injection. Cyclosporine was originally brought to market by Sandoz, now Novartis, under the brand name Sandimmune in 1983. It had variable bioavailability among patients with diarrhea, biliary diversion, diabetic gastroparesis, or malabsorption secondary to its high dependence on bile solubility [2]. This was addressed by the introduction of a microemulsion formulation, Neoral [3, 4] (Novartis), in July 1995. Several other similar cyclosporine formulations have subsequently been introduced in the market, and a timeline is summarized in Table 1.

Neoral, as the branded product, has been the favored choice of physicians for many years despite facing competition from the available generics. The approval process for generics was simplified after introduction of legislation in 1984 (commonly known as the Waxman-Hatch amendments). The process, also known as “Abbreviated New Drug Applications” (ANDA) has helped to increase availability of generic—or AB-rated—equivalents. The current FDA standard of AB rating indicates that bioequivalence has been studied and demonstrated and is the standard mandated criteria for generic formulations of all prescription drugs. 

Generics are tested against Brand in healthy young volunteers by giving a single dose of the reference formulation and the generic formulation that is being tested. Bioequivalence studies are performed using AUC (area under the time-concentration curve) and *C*_max⁡_ (maximum plasma concentration) to demonstrate that the generic has similar pharmacokinetics as the brand formulation. AUC serves as a surrogate for the extent of absorption whereas the *C*_max⁡_ and the time of its occurrence (*t*_max⁡_) together characterize the rate of absorption [5–7]. It is concluded that two pharmaceutical agents are not different from one another if the 90% confidence interval of the ratio of a log-transformed exposure measure (AUC and/or *C*_max⁡_) falls within the range 80–125%. Unless otherwise indicated by a specific guidance statement, the traditional bioequivalence limit of 80 to 125 percent is the same for nonnarrow therapeutic range drugs and for narrow therapeutic range drugs. The 80–125% bioequivalence acceptance range translates into a difference of −20 to +25% in the rate and extent of absorption between two drug products for a single dose exposure. Standard bioequivalence criteria do not require the generic formulation to be evaluated in target patient populations, over repeated exposure, in unhealthy people, or by administration intravenously. In addition, generics are not tested against other generics.

Some physicians and patients have raised concerns over generic versions of critical drugs by claiming a difference in quality and therapeutic efficacy compared to the brand name drug [7–9]. The arguments given are as follows.

1. The FDA acceptance limits for generics (80–125%) is too broad as there is a potential difference of as much as 45%.
2. Generic drugs are tested only in healthy volunteers and may act differently in the target disease population, resulting in uncontrolled clinical risks.

Every transplant physician encounters situations where a choice between brand versus generic formulation arises for financial or insurance reasons. For narrow therapeutic index drugs, this decision becomes more critical. The FDA defines these products as “those containing drug substances that are subject to therapeutic drug concentration or pharmacodynamic monitoring, and/or where product labeling indicates a narrow therapeutic range designation” [10]. As cyclosporine is a critical dose/narrow therapeutic index drug, a change in drug level has the potential to either cause rejection or result in renal toxicity depending on low or high levels, respectively.

Many pharmacies in the United States are increasingly substituting micro emulsion forms of cyclosporine, either by mandate or by choice. State regulations vary on the requirement of pharmacists to notify primary care providers of generic cyclosporine substitution, and subsequent additional therapeutic drug monitoring to verify consistency in drug exposure is frequently not undertaken. This, in turn, often leads to doubts in the physician's as well as the patient's mind, and ultimately leads to specifying “DAW” (dispense as written) on the hand-written prescription. 

One of the reasons given for the prospering generic market is healthcare dollar savings. It has been estimated that use of generics instead of brand name drugs in the year 2000 in America alone would have saved $8.8 billion, equivalent to 11% of total drug expenditure [11]. Another explanation for the prospering generic market is improved compliance with less expensive medications, for obvious reasons. However, in regards to cyclosporine generics, there have been concerns about the savings from their use in transplant patients. The argument given is that upfront savings are offset by the cost incurred by rejection of grafts, frequent monitoring of drug levels, or even the increased dosage requirement for the drug [12].

The purpose of this discussion is to review evidence for and against equivalence between brand and AB-rated equivalent cyclosporine, and thereby assist the transplant community in making knowledgeable decisions.

## 2. Supporting Data

There is scarcity of studies comparing Neoral with generic cyclosporine. In our literature search we came across fewer than half a dozen published articles addressing this important issue. Several studies have been reported from European countries claiming equivalence of generic and brand cyclosporine [13, 14] while one from a US center has suggested more frequent graft rejection on generic Gengraf [15]. The existing package insert for Neoral includes data related to limited comparisons with generic Sandimmune for transplant, rheumatoid arthritis, and psoriasis patients. In general, Neoral had increased bioavailability when compared to Sandimmune in small absorption studies. The mean cyclosporine area under the curve (AUC) and peak blood cyclosporine concentration (*C*_max⁡_) were both higher for Neoral, but dose-normalized trough concentrations were similar for both formulations. The percentage of dose present as major metabolites was also similar in both preparations.

The main backbone of generic formulations—bioequivalence—has been questioned in some studies. The inability of standard testing to confirm bioequivalence in a transplant population was demonstrated in the case of the SangCyA formulation of CsA. SangCya had a Class II recall by FDA, issued on products that have a low chance of causing major injuries or death, but where there is still the possibility of serious adverse events with irreversible consequences. It was voluntarily withdrawn from the market in 2000 after initial approval in 1998 following successful bioequivalence studies in healthy volunteers. It was found that bioavailability for SangCya was significantly different in comparison to Neoral when consumed with apple juice [16]. This finding highlighted the fact that known and unknown variables, even something as innocuous as coadministration of apple juice, may create potential confusion.

In the solid organ transplant setting, additional limitations in regards to bioequivalence testing have given rise to concerns [17]. Cyclosporine absorption may differ between healthy volunteers and transplant recipients [6]; indeed, absorption variability in transplant recipients has been documented to be related to time after transplantation and the type of organ graft [18, 19]. In addition, patient characteristics such as age [20, 21], ethnicity [22, 23], or comorbid disease [24, 25] may affect cyclosporine absorption.

Isolated reports have been published that other immunosuppressive medication absorption, including sirolimus, may be affected by generic versus brand cyclosporine formulations [26]. Intestinal drug-drug interactions are a general problem in transplantation and can occur when foods, herbal drugs, and other drug formulations are taken at the same time as the immunosuppressant. This issue is obviously not unique to either brand or generic drugs.

Currently, there is a paucity of data relating to the efficacy and safety of transferring transplant patients from reference Neoral formulations to a generic formulation or, indeed, from one generic form of cyclosporine to another. There are few studies evaluating pharmacokinetics or clinical outcomes between the cyclosporine microemulsion reference drug (Neoral) and AB-rated bioequivalents (namely, Gengraf). The following two studies have assessed the effect of transferring stable renal transplant patients from the Neoral to a generic formulation (Gengraf) of cyclosporine. 

Roza et al. [27] switched 50 stable renal allograft recipients in a multicenter, US-based study, from Neoral to Gengraf and then back to Neoral over 35 days on a dose-for-dose basis. This trial found no significant difference in the mean pharmacokinetic measurements during the three periods of the study, and there was no need for dose adjustment in any patient. The authors concluded that the pharmacokinetics of Gengraf were equivalent to those of Neoral. No statistically significant differences in *C*_max⁡_, AUC0 → 12 hr, *C*_min⁡_, and T max between Neoral at day 14 and Gengraf at day 28 were observed. However, only mean values of pharmacokinetic parameters were provided (*C*_max⁡_, trough blood concentration (*C*_0_), AUC, and time to maximum blood concentration (*t*_max⁡_)), without any individual data or ranges.

A second study by Carnahan and Cooper [28] involved 41 stable kidney transplant patients in an open-label clinical trial. The trial was conducted primarily to assess differences in steady-state cyclosporine concentration and serum creatinine after conversion from Neoral to Gengraf. Secondary goals were to evaluate changes in cyclosporine dosing regimen, Cyclosporine toxicity, graft rejection, hospital/emergency room admission, and changes with medications which could interact with cyclosporine. The authors reported no untoward incidents after an average of 18 weeks. 

The FDA acknowledges the limitations of bioequivalence studies and recognizes the need for increased assurance of the interchangeability for drug products containing narrow therapeutic index (NTI) drugs. FDA suggests additional testing and/or controls to ensure the quality of drug product containing immunosuppressant [10].

Concerns were raised in a study by Qazi et al. [29] where 6-month postrenal transplant patients with stable graft function were randomized to either remain on Neoral (*n* = 9) or switch to Gengraf (*n* = 73). Serum creatinine and cyclosporine troughs were measured at baseline and 2 weeks after the switch. Of the studied patients, 13 (18%) required a dose change after transfer to Gengraf. The mean CsA trough level in all patients converted to Gengraf rose from 180.5 ± 8.4 ng/mL to 195.0 ± 9.8 ng/mL (*P* < 0.05), which was statistically (but not necessarily clinically) significant. Interestingly, the mean baseline CsA level was 234 ± 96 ng/mL, rose to 289 ± 102 ng/mL after conversion, and fell back to 239 ± 151 after decreasing the dosage. The mean serum creatinine did not rise significantly. No dose changes were required among the patients who remained on Neoral. In summary, the Qazi study of 82 stable renal transplant recipients at least 6 months after transplant revealed that nearly 20% of patients who switched to a generic CsA preparation that was considered bioequivalent required a dose adjustment to return to the preconversion cyclosporine trough level. The potential for adverse effects resulting from such conversions (especially in patients who are seen only once in several months) and when the patient or the transplant care providers are unaware of the switch is of considerable concern, but true outcome data are still not available.

In 2006, Hibberd et al. [30] reported a study from Australia comparing Cysporin (generic) versus Neoral kinetics. The pharmacokinetic profile of Cysporin and Neoral were found to be different: for Cysporin the extent of absorption was lower and the rate of absorption was slower than that for Neoral. The authors felt that a trial in transplant recipients (not healthy volunteers) is needed to determine the pharmacokinetics and bioequivalence of a generic immunosuppressant, particularly a critical concentration-time profile drug such as cyclosporine A. 

In 1999, the National Kidney Foundation published a White Paper consensus [31] document to suggest recommendations for the safe and effective use of generic immunosuppressants based on the expert opinion of a multi-disciplinary group of participants and their review of the literature. In summary, recommendations for improving the FDA approval standards for generic immunosuppressants included the following.

1. Defining critical-dose drug characteristics and inclusion of immunosuppressive agents such as cyclosporine and tacrolimus in the list of critical-dose drugs.
2. Need for replicable pharmacokinetic studies of critical dose drugs as part of the approval process for both the innovator drugs as well as subsequent generics.
3. Bioequivalence studies in subpopulations of transplant patients (e.g., pediatric, African-American, or diabetic patients).

Further recommendations were made pertaining to safe and effective use of generic immunosuppressant agents, including the following.

1. Patient education and involvement in decision making before any switch from brand to generic and also from one generic to another.
2. Consistency in state regulations for pharmacist to notify the patient as well as physician prior to any substitution of an immunosuppressive medication.
3. Careful evaluation of bioequivalence data for drugs by physicians, so that appropriate prescribing decisions can be made related to generic substitution.
4. Consideration for appropriate drug monitoring techniques (including blood levels) if patients are switched from one formulation to another (e.g., brand to generic, generic to generic).
5. The importance of documenting and reporting adverse events with innovator and generic drugs.
6. Patient education to identify drug formulations and to alert the physician if a drug is substituted.

The American Society of Transplantation [32] published a similar report in 2003 on the use of generic immunosuppressants in the transplant settings. Recommendations are summarized as follows.

1. Need for consistency in use of selected immunosuppression formulation, timing of drug administration, and blood level monitoring.
2. Need for “pill and container” uniqueness among generic alternatives
3. Need for physicians and patients to be notified by pharmacists if there is any switch in dispensed brands
4. Need for patients to inform physician if any switch has taken place so that appropriate drug monitoring can be undertaken.
5. Incorporating bioequivalence studies in at-risk patient populations into generic drug approval process.

Most participants in the American Society of Transplantation forum [32] supported *de novo* usage of generic cyclosporine in low-risk patients or even a switch from brand to generic as long as the patient and care providers are clearly informed about the switch, so that when indicated additional tests could be performed to ensure desired drug levels. Because of insufficient bioequivalence data in the African American and pediatric population, generic substitution was not recommended in these groups.

Both AST and NKF recommendations for practice and policy seem rational and safe approaches while more definitive data are accumulated. The need for patient and provider education and awareness is clearly emphasized in these guidelines. In actual practice, however, changes often occur without the benefit of all relevant parties being involved in the decision making process. Pharmacists, providers, and patients are intricately linked in this important area, but often overlook the importance of two-way communication and followup when formulation changes are contemplated or actually undertaken. As such, the guidelines, although not new, are still relevant for safety but have not been incorporated into routine practice.

## 3. Summary

The best available evidence suggests that there are conflicting results as to whether a change from brand name product to generic equivalent will result in similar levels and outcomes. The inability to attain similar trough levels in a significant percentage of patients after a 1 : 1 switch is a concern, but this shortcoming can be easily corrected by ongoing therapeutic drug monitoring to ensure that the new steady states fall within an intended targeted range. Although this may offset some cost savings in the short term, the significant risk of jeopardizing graft function could be avoided. 

Cyclosporine is a narrow therapeutic-range drug for which small variations in exposure may have severe clinical consequences for transplant patients. In its Guidance for Industry on bioequivalence, the FDA recommends “that sponsors consider additional testing and/or controls to ensure the quality of drug products containing narrow therapeutic range drugs. The approach is designed to provide increased assurance of interchangeability for drug products containing specified narrow therapeutic range drugs.” However, FDA guidance does not elaborate what “additional testing and/or controls” could or should be carried out to assure the interchangeability for drug products [10].

Currently, there is a lack of comparative outcome data relating to the pharmacokinetics of the reference formulation Neoral and generic formulations in transplant recipients. Prospective clinical studies investigating the efficacy and safety of generic formulations in both *de novo* and long-term transplant patients are also awaited. For drugs such as cyclosporine, which exhibit complex absorption patterns and for which maintaining therapeutic exposure levels is critical to patient wellbeing and survival, the transplant physician must scrutinize the available pharmacokinetic and clinical data carefully before prescribing new generic formulations.

Until further evidence is available on the transfer of transplant patients to or between generic formulations of cyclosporine, any transfer to a different cyclosporine formulation should be undertaken with close supervision. The best available information to date, however, does not support the frequently held but unsubstantiated belief that generic preparations of immunosuppressive drugs are not as effective as brand names or that conversion from brand to generic is associated with significant danger.

## Figures and Tables

**Table 1:** Cyclosporine development timeline.

Date	Significant event
November 1983	Sandimmune (cyclosporine) oral solution approval
March 1990	Sandimmune (cyclosporine) capsule approval
July 1995	Neoral capsules and solution approval (brand cyclosporine modified)
October 1998	SangStat's SangCya (generic cyclosporine modified) approval
January 2000	Cyclosporine modified capsules (Sandoz) approval
May 2000	Cyclosporine modified capsules (Gengraf, Abbott)
December 2000	SangCya withdrawal
December 2000	Cyclosporine modified capsules (Pliva) approval
December 2001	Cyclosporine modified solution (Pliva) approval
May 2002	Cyclosporine capsules (generic Sandimmune, Apotex) approval
September 2004	Cyclosporine solution (generic Sandimmune, Morton Grove) approval
January 2005	Cyclosporine modified solution (Novex) approval
March 2005	Cyclosporine modified capsules and solution (Ivax) approval
